# Integration of clinical parameters, genotype and epistaxis severity score to guide treatment for hereditary hemorrhagic telangiectasia associated bleeding

**DOI:** 10.1186/s13023-020-01453-1

**Published:** 2020-07-13

**Authors:** Joan D. Beckman, Quefeng Li, Samuel T. Hester, Ofri Leitner, Karen L. Smith, Raj S. Kasthuri

**Affiliations:** 1 Department of Medicine, Division of Hematology, Oncology, and Transplantation, University of Minnesota, Minneapolis, MN 55455 USA; 2 Department of Biostatistics, University of North Carolina at Chapel Hill, Chapel Hill, NC USA; 3 Department of Internal Medicine, University of North Carolina at Chapel Hill, Chapel Hill, NC USA; 4 Department of Genetics, University of North Carolina at Chapel Hill, Chapel Hill, NC USA; 5 Department of Medicine, Division of Hematology/Oncology, University of North Carolina at Chapel Hill, CB#7035, 8206B Mary Ellen Jones Bldg, 116 Manning Drive, Chapel Hill, NC 27599 USA

**Keywords:** Telangiectasia, hereditary hemorrhagic, Anemia, Epistaxis, Osler-Rendu-weber disease, ACVRL1 protein, human, Endoglin, Iron, Antifibrinolytic agents

## Abstract

**Background:**

Hereditary Hemorrhagic Telangiectasia (HHT) is a rare inherited disorder characterized by development of mucocutaneous telangiectases and visceral organ arteriovenous malformations, which can lead to recurrent, spontaneous bleeding and development of iron deficiency anemia. The primary objective of this study was to ascertain the relationship between epistaxis severity scores (ESS), laboratory values, genotype, and phenotype in HHT. Our secondary objective was to assess efficacy of systemic antifibrinolytic therapy in reducing ESS in HHT.

**Methodology:**

We conducted a retrospective review of patients seen at the UNC HHT Center from January 1, 2009 to February 28, 2015. ESS, demographics, and results of genetic testing were abstracted from the medical record. Response to antifibrinolytic therapy was evaluated by comparing pre-post ESS.

**Results:**

One hundred and forty nine patients were eligible with 116 having genetic testing and 33 without. Age, hemoglobin and ferritin levels were predictive of ESS. Of the 116 patients that underwent genetic testing: 63 had an *ACVRL1* mutation, 40 had an *ENG* mutation, 2 had a *SMAD4* mutation, and 11 patients had no pathologic HHT genetic variation detected. Compared to patients without a detectable HHT-associated genetic variation, patients with a HHT-associated genetic variation had higher ESS scores (*p* < 0.05). Neither ESS nor genotype was predictive of pulmonary or brain AVMs. Twenty-four HHT patients with ESS > 4 were started on antifibrinolytic therapy (tranexamic acid or aminocaproic acid) and had a post-treatment ESS recorded. All patients had a decrease in ESS of > 0.71 (minimal meaningful difference), but patients taking antifibrinolytics displayed larger decreases. No patients on antifibrinolytics experienced a VTE with median follow up of 13 months.

**Conclusions:**

We demonstrate that the ESS correlates with age, hemoglobin and ferritin. Additionally, we demonstrate that HHT patients with genetic mutations have higher ESS scores. Our data demonstrate that antifibrinolytics are effective in decreasing epistaxis severity and safe with long-term use in HHT patients.

## Background

Hereditary Hemorrhagic telangiectasia (HHT), also known as Osler-Weber-Rendu disease, is a rare autosomal dominant vascular disorder that manifests as multiple mucocutaneous telangiectases, classically in the nose, lips, fingers, hands and the gastrointestinal (GI) mucosa [1]. Affected individuals may also develop visceral arteriovenous malformations (AVMs) in the brain, lung, or liver [1]. Clinically, the diagnosis of HHT is made based on the Curacao criteria, which include: (i) spontaneous and recurrent epistaxis, (ii) mucocutaneous telangiectasias, (iii) family history of HHT in a first-degree relative, and (iv) visceral AVMs [2]. Individuals with ≥3 criteria are categorized as definite HHT, 2 criteria as possible HHT and ≤ 1 as HHT unlikely. A majority of HHT patients have mutations in either endoglin (*ENG,* chromosome 9q34) or activin A receptor type II-like 1 (*ACVRL1/*ALK1, chromosome 12q13), both of which are components of the transmembrane receptor complex for TGF-β pathway ligands [1, 3, 4]. Mutations in *SMAD4* are the cause of HHT in a small number of patients (3–5%), and they develop juvenile polyposis in addition to HHT [5]. A pathogenic gene variant is expected to be identified in around 85% of patients with HHT, and genetic testing will be negative for such a mutation in about 15% of individuals with clinically diagnosed HHT [6, 7].

Clinically, the most common HHT manifestation is epistaxis, which occurs in > 90% of patients [8]. Epistaxis can be associated with significant morbidity and decreased quality of life related to iron deficiency anemia, need for chronic iron infusions and, in severe cases, blood transfusions [8–10]. Until 2010 clinical assessment and surrogate markers, such as iron deficiency +/− anemia, were used to assess epistaxis severity. The development of the epistaxis severity score (ESS) in 2010 was a significant advance in this regard [11]. The ESS is a weighted questionnaire that evaluates various characteristics associated with epistaxis severity. While the ESS has recently been validated, it does have some drawbacks [10, 12]. First, it includes assessment for presence of anemia (history and need for transfusions) but does not specify the cause of anemia. As it was developed for use in patients with epistaxis, this is presumed to be the cause. Second, information regarding need for iron replacement therapy is not included in the ESS. Of note, the ESS does not incorporate information regarding HHT genotype given limited data on the relationship between HHT genotype and epistaxis frequency/severity [6, 13–15]. The interactions between the ESS, HHT genotype and other disease manifestations, such as visceral AVMs, has not yet been explored.

Historically, management of epistaxis was primarily orchestrated by Otolaryngologists as surgical interventions, such as cauterization (silver nitrate or electrocautery), were the cornerstone of epistaxis management [16, 17]. Recently, approaches like laser ablation of mucosal telangiectasias have supplanted cauterization at tertiary referral centers and HHT centers of excellence. The importance of preventive and pharmacological approaches for management of epistaxis is increasingly appreciated [18]. Pharmacological agents, such as antifibrinolytic and antiangiogenic agents, have now been evaluated for epistaxis management in HHT [19–24]. While the international guidelines for the diagnosis and management of HHT do not recommend use of antifibrinolytics for patients with mild to moderate epistaxis, these recommendations are based on limited and anecdotal data which lacked standard characterization of epistaxis severity. Recent studies have demonstrated antifibrinolytics to be safe and effective for management of epistaxis in patients with HHT [20, 21, 25]. However, these studies had relatively short follow up (average 3 months), and the utility and safety of long-term antifibrinolytic therapy for treatment of epistaxis in HHT is not known.

Standardized symptom assessment tools, such as the ESS, are useful in providing objective appraisal of subjective symptoms; however, correlation with quantitative clinical parameters is important if these tools are to be used to guide patient management. Our retrospective, single center study had several objectives: (i) to ascertain the relationship between specific laboratory parameters and the ESS; (ii) to evaluate genotype-phenotype interactions with regards to epistaxis severity; (iii) to evaluate whether the ESS can be used to guide therapeutic decisions; and (iv) to evaluate whether prolonged treatment with antifibrinolytic therapy would be efficacious and safe in patients with HHT.

## Results

### ESS correlates with age and hemoglobin

**Fig. 1:**
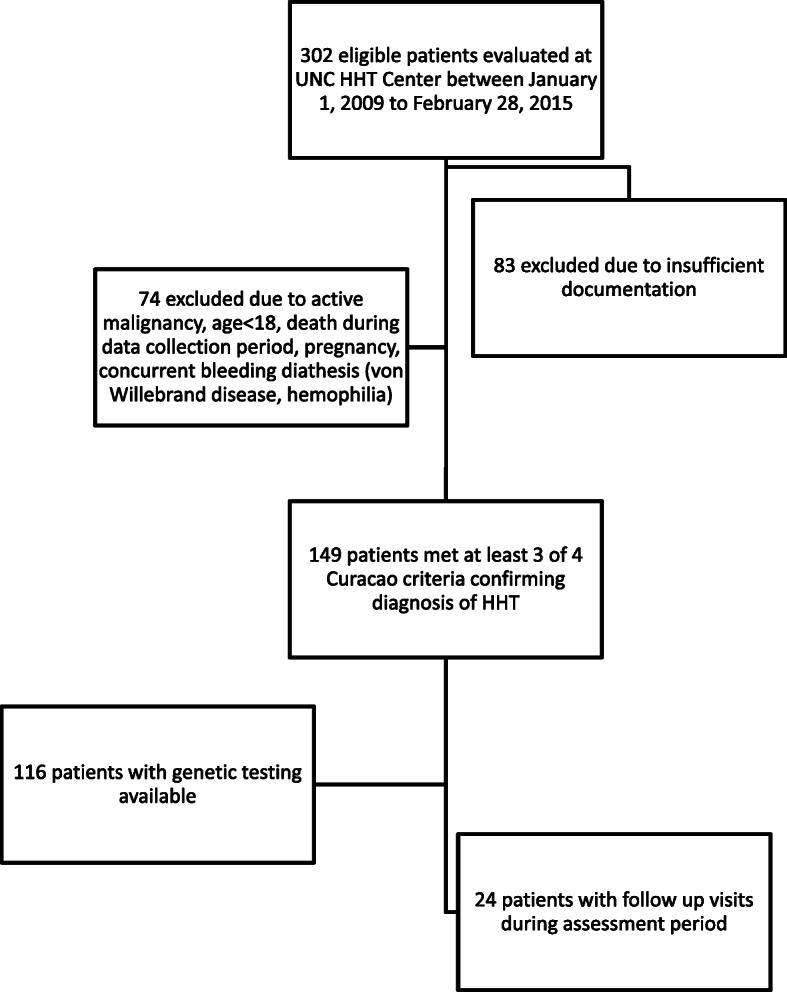
Patient selection chart

**Fig. 2:**
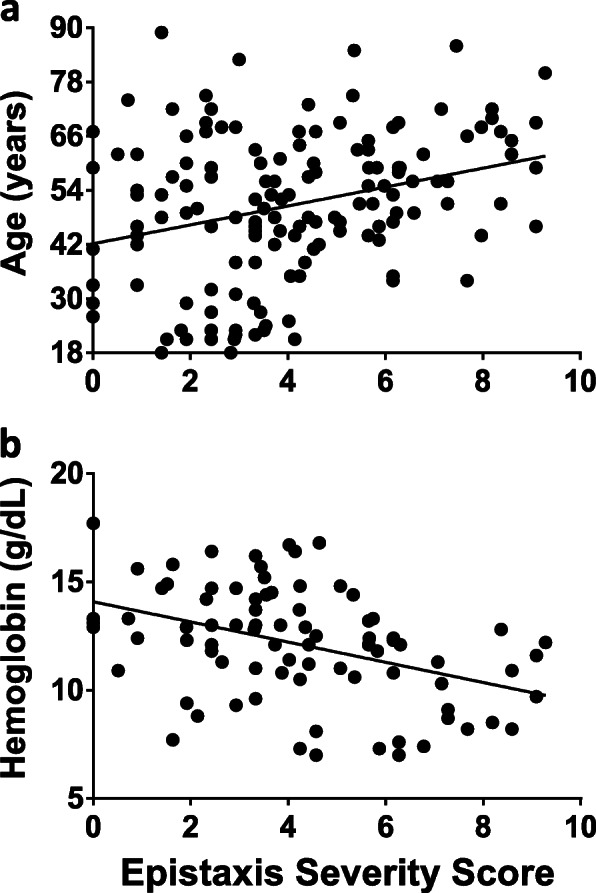
ESS correlates with age and hemoglobin. **(a)** Age is positively correlated with ESS score. *P* < 0.05 via Pearson correlation. **(b)** Hemoglobin (*P* < 0.02) is negatively correlated with ESS score via multivariate linear regression

One hundred and forty nine adults were eligible for study evaluation (Fig. 1). Table 1 summarizes the demographic characteristics of study participants. Epistaxis was the primary clinical symptom of HHT, consistent with previous finding that ~ 50% of patients developing epistaxis prior to 20 years of age and > 90% endorsing epistaxis by age 45 [26]. To identify if other demographic or clinical parameters could predict ESS severity, we evaluated the correlation between ESS and available demographic and clinical parameters. Consistent with the literature, age positively correlated with increased ESS (*p* < 0.0002, Fig. 2a). We used a multivariate linear regression model to regress ESS on gender, race, hemoglobin (Hgb), hematocrit (Hct), mean corpuscular volume (MCV), mean corpuscular hemoglobin (MCH), mean corpuscular hemoglobin concentration (MCHC), iron therapy, total iron binding capacity (TIBC), iron saturation, ferritin, and HHT genotype. To avoid overfitting, we employed the stepwise forward-selection-backward-elimination technique to select the best subset of variables predictive of ESS. This method identified Hgb and ferritin as independent predictors of a higher ESS, with both variables significantly inversely associated with increased ESS (*p*-value for Hgb = 0.019, *p*-value for ferritin = 0.043; Fig. 2b). Collectively, these data demonstrate that increased age, low Hgb and low ferritin are associated with more severe epistaxis.

**Table 1:** Baseline Characteristics of HHT Patient Cohort

Clinical Variables	Reference Range	Mean ± Standard Deviation or n (%)
*Race*		
Caucasian		134 (89.9)
Non-Caucasian		15 (10.1)
*Sex*		
Male		91 (61.1)
Female		58 (38.9)
Age, y		50.6 ± 16.1 (range 18–89)
Epistaxis Severity Score		4.03 ± 2.34
Hemoglobin (Hgb, g dL^−1^)	13.5–17.5	12.12 ± 2.62^a^
Hematocrit (Hct, %)	36–46	37.4 ± 6.76^b^
Mean Cell Volume (MCV, fL)	80–100	85.38 ± 8.24^c^
Mean Corpuscular Hemoglobin Concentration (MCHC g dL^− 1^)	31–37	32.4 ± 1.97^b^
Iron (μg dL^− 1^)	35–165	63.10 ± 47.8^d^
Transferrin (Tfn, mg dL^− 1^)	200–380	307.42 ± 54.43^e^
Total iron-binding capacity (TIBC μg dL^− 1^)	252–479	383.08 ± 69.27^f^
Iron saturation (%)	20–50	17.73 ± 15.16^e^
Ferritin (ng mL^− 1^)	27–377	29.31 ± 49.96^g^

Number of patients is *n* = 149, unless denoted by footnote as below

^a^*n* = 65

^b^*n* = 73

^c^*n* = 66

^d^*n* = 85

^e^*n* = 88

^f^*n* = 87

^g^*n* = 76

### ESS higher in individuals with detectable HHT-associated genetic variations

**Fig. 3:**
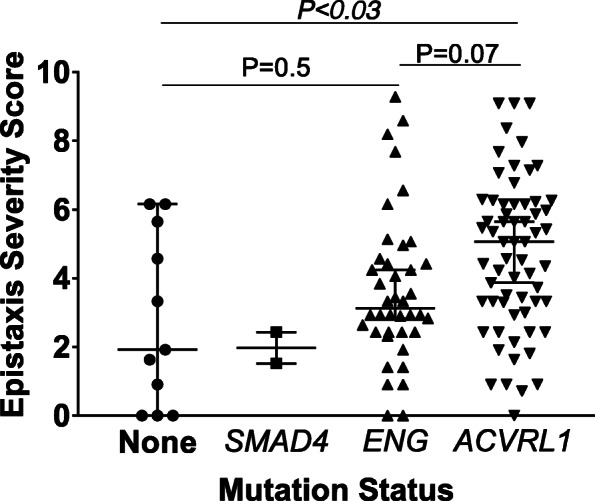
ESS higher in patients with detectable HHT-associated genetic variations. All patients met Curacao criteria for HHT. Compared to patients lacking a detectable HHT-associated mutation (*n* = 11), patients with an *ACVRL1* (*n* = 63) have a significantly higher ESS. Patients with an *ENG* (*n* = 40) displayed a trend toward higher ESS. Values represent median ± 5–95% confidence interval, *p*-values determined by one-way ANOVA with Sidak post-hoc test

HHT genetic testing data was available in a majority of eligible patients (116/149, 77.8%, Fig. 1). The prevalence of detectable HHT-associated genetic variations were as follows: *ACVRL1* = 63 (54.3%), *ENG* = 40 (34.5%) and *SMAD4* = 2 (1.7%). Additionally, 11 patients (9.5%) met the consensus diagnostic criteria for HHT (at least three Curacao criteria) and carried a HHT clinical diagnosis but did not have detectable genetic variations in *SMAD4, ACVRL1* or *ENG*. Compared to individuals in whom the HHT diagnosis was made on clinical grounds (i.e. no known HHT-associated pathogenic variations on genetic testing), individuals with a detectable HHT-associated genetic variation have a trend toward higher ESS scores (median 1.9 [0–6.2, 95% confidence interval, CI,] versus 4.1 [3.3–4.6, 95% CI], *p* = 0.08). Regarding the different HHT genotypes, individuals with genetic variations in *ACVRL1* had a significantly higher ESS compared to patients without a mutation in *ACVRL1* (median 5.1, [3.9–5.7 95% CI,] *p* < 0.03, Fig. 3). Further, compared to the ESS of individuals with genetic variations in *ENG* (median 3.1 [2.8–4.2 95% CI]), individuals with *ACVRL1* mutations had a trend for higher ESS (*p* = 0.07). We were unable to make comparisons for individuals with *SMAD4* mutations given the small number of patients in our study cohort. Collectively, these results indicate that detection of an HHT-associated gene variation portends more severe epistaxis.

### ESS and genotype do not predict visceral AVMs

Given the observed association between ESS and HHT genotype, we next evaluated whether prevalence of brain or pulmonary AVMs correlated with ESS and/or HHT genotype. Seventy eight patients had data regarding brain AVMs and 79 had data regarding PAVMs. Brain AVMs were present in 13 patients (16.7%, 13/78 screened) and PAVMs were present in 43 patients (54.4%, 43/79 screened). With regard to mutations, 1 patient with a brain AVM had a detectable HHT-associated mutation and 41 patients with PAVMs had a detectable HHT-associated mutation. Using both chi-square testing and regression analysis we were unable to discern an association between gender or presence of a detectable HHT-associated mutation and the presence of either brain or pulmonary AVMs. Likewise, when compared to patients without brain or pulmonary AVMs, there was no significant difference between median ESS in patients with either brain AVMs (median 4.1 [3.3–5.1 95% CI] verses 5.0 [2.4–6.3 95% CI]) or PAVMs (median 5.1 [4.0–5.7 95% CI] verses 3.3 [2.8–4.2 95% CI]). Combined, these data demonstrate that gender, genotype, and ESS are not associated with presence of brain or pulmonary AVMs.

### ESS higher in HHT patients with iron deficiency anemia

**Fig. 4:**
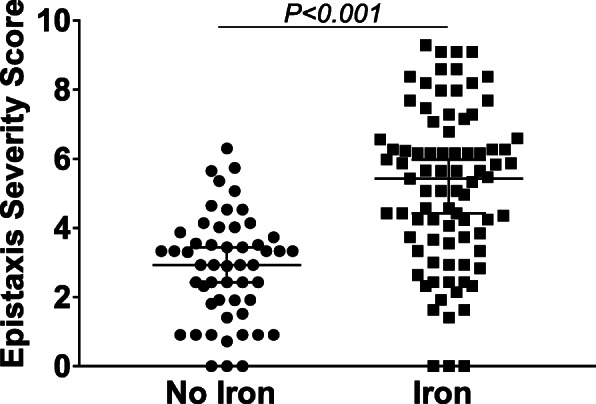
HHT patients on iron therapy have increased ESS. Removing patients with history of GI bleeding, HHT patients not on iron therapy (*n* = 52) had a significantly lower ESS compared to HHT patients on iron therapy (*n* = 83). Iron therapy includes oral iron, IV iron or regular red blood cell transfusions. Values are median ± 5–95% interval, *p*-value determined by student’s t-test

The ESS assessment includes evaluation for presence of anemia and need for interventions (i.e. blood transfusions) as the primary causes of iron deficiency anemia in HHT patients are epistaxis and GI bleeding. However, as the presence of GI bleeding is not assessed in the ESS, the ESS may not accurately reflect anemia due to epistaxis alone. Given these considerations, we eliminated patients in our cohort who had history of significant GI bleeding (*n* = 14) to evaluate the relationship between need for iron therapy and/or chronic transfusions and ESS. Compared to HHT patients not on iron (*n* = 52), patients on iron therapy (oral and/or parenteral iron) or intermittent blood transfusions (*n* = 83) had a significantly (*P* < 0.0001) higher ESS (median 2.9 [2.4–3.4 95% CI] versus 5.4 [4.4–6.0 95% CI], Fig. 4). There was no association between need for iron therapy and HHT genotype (data not shown). These data illustrate that the ESS accurately reflects the contribution of epistaxis-derived blood loss to chronic iron deficiency anemia in patients with HHT.

### ESS to guide and monitor therapy

**Fig. 5:**
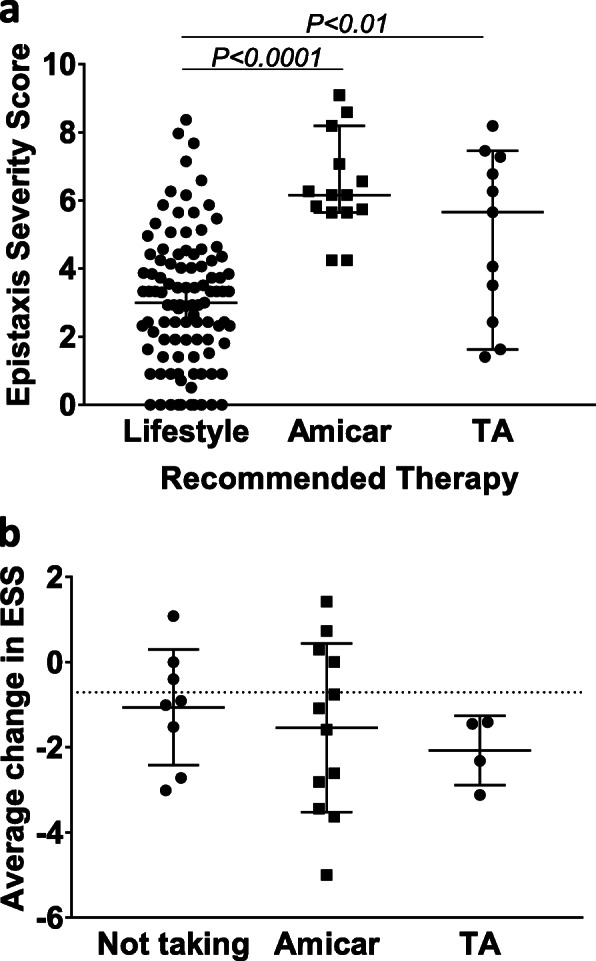
Patients prescribed pharmacologic therapy have higher ESS, which is reduced with antifibrinolytic therapy over time. **(a)** Patients not assigned to pharmacologic therapy (*n* = 101) have significant lower ESS compared to patients assigned to either aminocaproic acid (Amicar, *n* = 14) or tranexamic acid (TA, *n* = 11). Values represent median ± 5–95% confidence interval, *p*-values determined by one-way ANOVA with Sidak post-hoc test. **(b)** Follow up was obtained on 24 patients with median time of 14.5 months. The minimal important difference in ESS is considered to be − 0.71 as noted by dashed line. All patients had a decrease in ESS over time. Compared to patients not on therapy (*n* = 8), patients whom took either amicar (*n* = 12) or tranexamic acid (*n* = 4) had larger reduction of ESS. Values are represented as median ± 95% CI

As HHT patients on iron therapy had increased ESS values, we next assessed if there was an association between increased ESS and specific therapeutic interventions. First, we evaluated which patients were likely to receive therapy. Overall, a majority (*n* = 101) of patients had an ESS score in mild-moderate category (ESS 3.0, 95% CI 2.4–3.4). All patients were advised to initiate lifestyle modifications and topical moisturizers (Fig. 5a). Patients with ESS above 4 were more likely to be assigned antifibrinolytic therapy. The median ESS among patients prescribed aminocaproic acid (*n* = 14) was 6.2 (5.7–8.2, 95% CI) and for patients prescribed tranexamic acid (*n* = 11) was 5.7 (1.6–7.7, 95% CI). Twenty four patients were followed with a median time between visits of 14.5 months (range 3–33 months). Patients with mild ESS score prescribed moisturizers and lifestyle modifications or who were not taking antifibrinolytics (*n* = 8) had a median decrease in ESS of − 0.96 (95% CI 1.1- -3.0) (Fig. 5b). Patients with moderate-severe ESS prescribed antifibrinolytics also decreased their ESS (median − 1.34, 95% CI 0.3- -3.4 for aminocaproic acid and − 1.9, 95% CI -1.4- -3.1 for tranexamic acid, *n* = 12 and *n* = 4, respectively, Fig. 5a). It was recently demonstrated that the minimum meaningful difference in the ESS is a score decrease of 0.71 [12, 27]. Based on this, patients in all ESS groups in our cohort demonstrated a significant improvement in epistaxis with the prescribed interventions (dotted line, Fig. 5b). Collectively, these results suggest that ESS can be used to both guide therapeutic interventions as well as monitor therapeutic response in HHT patients.

## Discussion

While phenotypic heterogeneity is well described in patients with HHT, epistaxis develops in the majority of patients (> 90%) by age 45 [26]. The severity of epistaxis can lead to medical complications like severe iron deficiency anemia and adversely affect patient quality of life [9, 10, 28]. Standardized symptom assessment tools, such as the ESS, are useful in providing objective appraisal of subjective symptoms. However, correlation with quantitative clinical parameters is important if these tools are to be used to guide patient management. Our study had several objectives: (i) to ascertain the relationship between specific laboratory parameters and the ESS; (ii) to evaluate genotype-phenotype interactions with regards to epistaxis severity; (iii) to evaluate whether the ESS can be used to guide therapeutic decisions; and (iv) to evaluate whether prolonged treatment with antifibrinolytic therapy would be efficacious and safe in patients with HHT.

The ESS is a weighted score for objective assessment of epistaxis severity. In our patient cohort, the ESS values were positively correlated with age, with older patients demonstrating higher scores. Additionally, variable selection to determine predictors of ESS found that both hemoglobin and ferritin were predictive of ESS. An age-dependent expression is well described in HHT and our findings are consistent with this observation. The finding that low hemoglobin and ferritin were predictors of higher ESS scores suggests epistaxis as the cause of iron deficiency anemia in our cohort. We omitted patients with known GI bleeding from this analysis given that GI bleeding could also cause anemia and the need for transfusions, thereby affecting 2 of 6 ESS parameters. Despite this, the association between ESS and need for iron supplementation remained, further confirming epistaxis as the major driver of iron deficiency anemia in our HHT cohort. Combined, these data provide independent validation of the use of ESS to assess epistaxis severity. However, in both the research and clinical settings, it is important to consider potential occult bleeding from GI telangiectases when administering and evaluating the ESS in patients with HHT.

The association between genotype, epistaxis, and visceral AVMs has been previously evaluated [6, 13, 14, 29–32]. Epistaxis is the most common (> 90%) manifestation in patients with both *ENG* and *ACVRL1* mutations, although patients with *ENG* mutations are believed to have onset of epistaxis at a younger age [6, 13–15, 33]. The adoption of the ESS allowed us the power to objectively evaluate the relationship between HHT genotype and epistaxis. We found that individuals with *ACVRL1* mutations demonstrated a trend (*p* = 0.07) towards higher ESS scores compared to those with *ENG* mutations. Interestingly, although number of individuals was low, we also found that patients with detectable HHT-associated genetic variations had higher ESS values compared to patients with a clinical HHT diagnosis but no detectable HHT-associated genetic variation. All patients in the clinically diagnosed HHT group (i.e. no detectable HHT-associated genetic variations) met stringent clinical diagnostic criteria for HHT [2]. Our findings raise the question of whether the clinical criteria lead to overdiagnosis of HHT in subjects with epistaxis. However, as our study and others highlight, there remains significant phenotypic heterogeneity in individuals with HHT, regardless of presence or absence of detectable HHT-associated genetic variants, and this needs to be considered when interpreting our findings. Larger studies comparing HHT patients with and without underlying pathogenic variants in HHT-associated genes are needed to further explore this finding. Overall, these data suggest that presence of a detectable HHT-associated genetic variation may be useful indicators of epistaxis severity over time.

The data regarding genotype and prevalence of pulmonary and cerebral AVMs varies between studies, with some studies demonstrating an association while others do not [6, 13, 14, 29, 31, 32, 34]. This discrepancy between studies may be related to racial/ethnic differences in the study populations, study methodologies, and the impact of co-existing gene variants that modify HHT biology [35, 36]. Our data do not demonstrate an association between gender or genotype and prevalence of pulmonary or brain AVMs; however, this may be due to lack of sufficient numbers, especially within the brain AVM cohort. Recent data from Mora-Lujan et al. has demonstrated an association between female gender and increased risk of hepatic AVMs [31]. Unfortunately, our data set did not include information regarding hepatic AVM. Additionally, with regard to pulmonary AVMs, our HHT Center considers patients as having a pulmonary AVM if they have pulmonary shunting on bubble study and/or presence of pulmonary AVMs on CT; other reported studies traditionally use presence of pulmonary AVM via CT to make the diagnosis. Given that previous work has indicated that patients with *ACVRL1* are less likely to have large pulmonary AVMs visualized by CT [14], our study may include patients as having AVMs that other studies would not.

A recent survey of worldwide HHT center screening practice patterns demonstrated wide variability regarding frequency of screening for pulmonary and other AVMs [37]. Given this variability, having data to stratify which HHT patients are at increased risk for cerebral, pulmonary or hepatic AVMs would be useful in standardizing care and developing guidelines. Therefore, we evaluated whether the ESS was useful in predicting presence of visceral AVMs. We did not find a correlation between ESS and the prevalence of visceral AVMs, although our study was underpowered for this analysis. Given our study and other recently published analysis, [31] a comprehensive meta-analysis of existing data may be useful to more definitively assess whether ESS correlates with prevalence/risk of visceral AVMs in patients with HHT.

Antifibrinolytic agents are commonly used for the prevention and treatment of mucocutaneous bleeding in patients with bleeding disorders. Previous publications have reported efficacy of systemic antifibrinolytics in the control of epistaxis in HHT. Although our study was underpowered to evaluate efficacy of antifibrinolytic therapy in the management of epistaxis in HHT, our findings confirm previous reports and is amongst the few to longitudinally apply the ESS to assess treatment response. A recent paper evaluated the ‘minimal important difference’ in the ESS as it pertains to improvement in patient quality of life. This was found to be a decrease in the ESS of 0.71 [12]. All patients treated with antifibrinolytics in our study demonstrated a decrease in ESS of > 0.71, suggesting that antifibrinolytics are an effective option for treatment of epistaxis in HHT. Further, our study also assesses the impact of non-pharmacological approaches for the management of epistaxis in HHT using the ESS. All patients with epistaxis were advised to use a humidifier overnight, topical moisturizers twice daily, and adopt lifestyle modifications deemed help decrease epistaxis risk. While compliance was not strictly assessed or enforced, even patients not treated with antifibrinolytics reported an improvement in ESS of > 0.71. This suggests that the ESS is a useful tool to objectively assess not only severity of epistaxis but also response to therapeutic interventions.

Previous reports on the use of antifibrinolytics in HHT were limited by a short duration of follow-up – only 3 months [20, 21, 25]. Thus the question of safety of long-term antifibrinolytic therapy remains unanswered. This becomes particularly important given the reported increased risk of VTE in HHT patients compared to the general population and the possible role of iron deficiency in determining thrombotic risk [38–40]. Although our retrospective study was underpowered to discern a significant benefit with the use of antifibrinolytics and lacked formal adverse events protocols to evaluate the safety and tolerance, our follow-up cohort included a subset that were on long-term (> 12 months) antifibrinolytic therapy. In our analysis we did not include individuals with history of VTE or VTE risk factors (i.e. pregnancy, active malignancy), but did include individuals with HHT and severe anemia who needed frequent blood transfusions or parenteral iron replacement. In our cohort, long-term oral antifibrinolytic therapy (both aminocaproic acid and tranexamic acid) was well tolerated. None of these patients developed a clinically significant PE or VTE while on this therapy. Despite stated limitations, we conclude that extended duration of antifibrinolytic therapy may be a safe and effective treatment option for epistaxis in patients with HHT.

The observational, retrospective design of our study creates several limitations. First, we lacked complete laboratory assessments in some patients, which limited the evaluation on severity of anemia. Second, genetic testing was performed at commercial labs. Therefore, in individuals for whom initial testing was negative, more detailed genetic analysis of UTR regions and sequencing for unidentified genetic variations was not performed; this would have been informative. Third, we acknowledge that in phenotype-genotype correlation studies, the influence of founder effect and referral bias is not insignificant as these may lead to a skewed or overrepresentation of specific genotypes within our patient cohort. However, the majority of our adult patients were unrelated. Finally, this study was neither prospective nor randomized, therefore patients were not on standardized doses of medications and clinicians were not blinded to treatment, which allows for bias. This also introduced variability in the dose, type and route of antifibrinolytic agents used.

## Conclusion

In conclusion, our retrospective study provides further evidence that ESS is a useful tool to assess severity of epistaxis in HHT. We demonstrate that the ESS predicts markers of iron deficiency anemia and that it can be used to both guide and monitor response to therapeutic interventions in patients with HHT. The ESS is thus a valuable tool both in patient care and for assessment of epistaxis in the research setting. Additionally, we demonstrate that ESS is not associated with presence of brain or pulmonary AVMs. Finally, in a small cohort of patients with follow-up data, we found the long-term use of antifibrinolytics to be safe and well tolerated.

## Methodology

### Cohort selection

University of North Carolina IRB approval was obtained to retrospectively review the medical records of adult patients with possible HHT who were evaluated at UNC HHT Clinic between January 1, 2009 to February 28, 2015. Three hundred and two patient encounters were identified. Seventy four patients with HHT were excluded because they met the following exclusion criteria: active malignancy, age < 18, death during data collection period, pregnancy, concurrent bleeding diathesis (von Willebrand disease, hemophilia). An additional eighty three were excluded from analysis due to insufficient documentation (Fig. 1). Therefore, 149 patients met at least three Curacao criteria confirming the diagnosis of HHT or had documented presence of a HHT-associated gene mutation.

### Epistaxis severity score

The ESS is a validated, weighted scoring system to objectively assess the severity of epistaxis in HHT [10, 11]. ESS scores documented in the patient charts were noted and confirmed based on documented patient histories. Data involving epistaxis duration, intensity and frequency from the patient history within the medical record were used to calculate an ESS in patients in whom no ESS was documented. If the ESS was not documented and could not be calculated based on available data, the patient was excluded from any analysis requiring the ESS (Fig. 1). For analysis evaluating the contribution of gastrointestinal bleeding history to ESS, patients with HHT were excluded if they had the following: documentation of recent (< 3 months) hospitalization for GI bleeding requiring transfusions or endoscopic intervention, history of inflammatory bowel disease, history of gastric varices.

### Genetic testing

Genetic testing information was available for 116 patients. Genetic testing was performed by either Ambry Genetics (Aliso Viegjo, CA, USA) or the University of Pennsylvania Genetic Testing Laboratory (Philadelphia, PA, USA) and involved evaluation for deleterious mutations in *ENG*, *ACVRL1* and *SMAD4*.

### Screening for solid organ AVMs

All patients underwent screening for visceral AVMs as per the HHT treatment guidelines [1]. Screening for brain AVMs was pursued with an MRI with and without contrast. Screening for pulmonary AVMs (PAVMs) was performed using an echocardiogram with saline contrast as the first step. All patients with a positive contrast echocardiogram proceeded to get a chest CT. A minority of patient proceeded directly to chest CT, either because of past history of pulmonary resection/embolization for PAVMs, history of a positive contrast echocardiogram in the past, or due to lack of local expertise with contrast echocardiography (when screening was pursued by local physician). PAVMs were considered present if the contrast echocardiogram was positive (regardless of subsequent chest CT findings) or if AVMs were noted on the CT scan in those patient where chest CT was the only imaging study performed for PAVM screening. Very small PAVMs can lead to positive contrast echocardiograms but not be visualized on chest CTs.

### Lifestyle modifications and antifibrinolytic therapy

All patients with HHT received instruction to initiate lifestyle modifications (use of humidifier overnight, avoidance of nasal trauma, dietary modifications to avoid foods and supplements that could cause platelet dysfunction and worsen epistaxis, such as omega-3 fatty acids, etc). Additional treatment with an antifibrinolytic agent was pursued in 48 patients. As the study was retrospective, the dose and type of antifibrinolytic was determined by preference of the provider, availability and patient tolerance. In all, one cohort (*n* = 14) was prescribed aminocaproic acid 1–3 g orally, one to three times a day titrated to symptoms. A second cohort (*n* = 11) was prescribed tranexamic acid 650–1300 mg two to three times daily and a third cohort (*n* = 15) was started on topical tranexamic spray (10% solution) 1 spray to each nostril twice daily (data not shown). A small number of patients (*n* = 8) were started on various combinations of oral and topical antifibrinolytic or oral antifibrinolytic plus laser ablation (data not shown).

Between 2009 and February 2015, 24 patients had follow up visits with a median time between visits of 14.5 months (range 3–33 months). As the 2016 NOSE trial failed to demonstrate a reduction in ESS with topical antifibrinolytics use, we excluded individuals on intranasal antifibrinolytic therapies [23]. Sixteen patients had started oral antifibrinolytic therapy, allowing for assessment of adverse events and repeat assessment of ESS after initiation of antifibrinolytic therapy.

### Statistics

Descriptive statistics (mean, median, standard deviation [SD], standard error of the mean [SEM], normality) were calculated using Graph Pad Prism v 7.02 (Synergy Software). Graph Pad and R version 3.2.2 was used for the data analysis. To select the covariates that best predicted ESS, a stepwise forward-selection-backward-elimination method was used. The two sample t-test was used to demonstrate the difference of median ESS scores with application of with Welch correction as appropriate. A one-way analysis of variance with Sidak (parametric) or Dunn’s (nonparametric) comparisons was used to evaluate differences of median ESS between patients with different HHT genotypes. The chi-squared test was used to determine association between genotype and the presence of either pulmonary or brain AVMs. Unless otherwise noted, *P < 0.05* was considered statistically significant.

## Data Availability

The datasets used and/or analyzed during the current study are available from the corresponding author on reasonable request.

## Abbreviations

AVM: Arterial venous malformation
ESS: Epistaxis severity score
HHT: Hereditary hemorrhagic telangiectasia
Hct: Hematocrit
Hgb: Hemoglobin
MCV: Mean corpuscular volume
MCH: Mean corpuscular hemoglobin
MCHC: Mean corpuscular hemoglobin concentration
PAVM: Pulmonary arterial venous malformation
TIBC: Total iron binding capacity
